# Association Between Controlling Nutritional Status (CONUT) Score and Retinopathy in Adults With Diabetes Mellitus: An NHANES Analysis

**DOI:** 10.1155/jdr/7303131

**Published:** 2025-09-27

**Authors:** Wen-Hsueh Chen, Kun-Lu Hsieh, Jau-Yuan Chen, Chao-Tung Chen

**Affiliations:** ^1^Department of Family Medicine, Chang Gung Memorial Hospital, Chiayi, Taiwan; ^2^Department of Family Medicine, Chang-Gung Memorial Hospital, Linkou Branch, Taoyuan, Taiwan; ^3^College of Medicine, Chang-Gung University, Taoyuan, Taiwan

**Keywords:** Controlling Nutritional Status (CONUT), diabetes mellitus (DM), diabetic retinopathy (DR), National Health and Nutrition Examination Survey (NHANES)

## Abstract

**Background:** Diabetes mellitus (DM) is a growing global health issue, with diabetic retinopathy (DR) being a significant complication causing vision loss. Whether the Controlling Nutritional Status (CONUT) score is predictive of DR has yet to be understood.

**Objective:** This study is aimed at exploring the relationship between CONUT scores and DR using national health data.

**Methods:** Data from adults aged ≥ 20 years diagnosed with DM were extracted from the National Health and Nutrition Examination Survey (NHANES) database from 2005 to 2016 and analyzed retrospectively. DR was identified based on participants' self-reported physician diagnosis. The CONUT score was calculated from serum albumin, lymphocyte count, and cholesterol levels, obtained from the NHANES lab profiles. Logistic regression analysis was used to determine the relationship between CONUT scores and the presence of DR. All data were analyzed in 2024.

**Results:** Data from 3494 NHANES participants (representing 17,619,250 people in the United States after weighting) were analyzed. After adjusting for relevant confounders, multivariable analysis revealed that each one-point increase in CONUT score was significantly associated with increased odds of DR by 11% (adjusted odds ratio [aOR] = 1.11, confidence interval: 1.02–1.22). Stratified analyses revealed significant associations between CONUT score and DR among patients with a DM duration of ≥ 10 years (aOR = 1.14, 95% CI: 1.03–1.26) and those younger than 60 years (continuous: aOR = 1.19, 95% CI: 1.03–1.37; high vs. low: aOR = 1.57, 95% CI: 1.05–2.35).

**Conclusions:** Poor nutritional status, indicated by higher CONUT scores, is associated with an increased likelihood of DR in adults with DM. The relationship was particularly evident among those with a DM duration of ≥ 10 years and younger than 60 years. These findings highlight the potential of CONUT score assessments as part of comprehensive diabetes care.

## 1. Introduction

Diabetes mellitus (DM) is a chronic metabolic disorder marked by elevated blood glucose levels, arising either from insufficient insulin production (Type 1 DM), resistance to insulin (Type 2 DM), or both [1]. It affects approximately 8.8% of adults worldwide, with estimates predicting a rise to 9.9% by 2040 [2, 3]. Proper disease management, which includes adherence to prescribed medications, dietary regulations, regular exercise, and diligent monitoring of blood glucose levels, can mitigate the risk of future complications. Failure to maintain these recommended lifestyle changes is strongly linked to an increased risk of severe complications, including diabetic retinopathy (DR), diabetic foot, and renal disease necessitating dialysis [4, 5]. DR remains a significant concern as it accounts for the majority of vision loss cases among individuals with chronic, poorly controlled diabetes, which highlights its impact as a major health issue [6, 7].

The Controlling Nutritional Status (CONUT) index is a comprehensive tool designed to assess protein reserves, immune competence, and caloric deficits using key parameters such as serum albumin levels, total lymphocyte count, and total cholesterol concentrations [8, 9]. The utility of the CONUT index extends beyond simple nutritional assessment. Recent studies have explored the prognostic applications of the CONUT score, analyzing its influence on posttreatment complications, clinicopathological variables, and long-term results in a range of diseases, including heart failure, liver disease, hypertension, and cancer [10–13].

Nutritional management is essential for DM patients. To date, the specific role of the CONUT score in patients with DR has not been clearly established. We hypothesize that poorer nutritional status, as indicated by a higher CONUT index, is associated with a greater likelihood of retinopathy in patients with DM. Thus, this study is aimed at exploring the relationship between the CONUT index and DR, utilizing data from a national database.

## 2. Methods

### 2.1. Data Source

This cross-sectional analysis utilized secondary data from the National Health and Nutrition Examination Survey (NHANES), conducted by the National Center for Health Statistics (NCHS), a division of the Centers for Disease Control and Prevention (CDC) in the United States. Available online at http://www.cdc.gov/nchs/nhanes/ is an ongoing series of yearly surveys that assess the health and nutritional status of both adults and children in the United States. It employs a sophisticated, multistage probability sampling design to ensure the data accurately reflect the civilian, noninstitutionalized US population. Researchers can access the data following approval from the NCHS. NHANES participants undergo a detailed household interview and a comprehensive health evaluation at a mobile examination center (MEC), which includes a physical examination, specific diagnostic tests, and various laboratory assessments. This thorough methodology ensures that the analysis of the NHANES data provides a robust and comprehensive evaluation of the US population's health on a national scale.

### 2.2. Ethics Considerations

The NHANES program is reviewed and approved by the NCHS Research Ethics Review Board, and all participants provide written informed consent prior to participation. As this study involved secondary analysis of publicly available, deidentified NHANES data, no additional ethical approval or informed consent was required. Further details regarding NHANES ethical oversight can be found on the NCHS website (https://www.cdc.gov/nchs/nhanes/about/erb.html). In accordance with NHANES data use guidelines, we conducted all analyses using anonymized data to ensure participant confidentiality and adherence to ethical standards.

### 2.3. Study Population

This cross-sectional study analyzed data from the NHANES from the dual-year cycles 2005–2006, 2007–2008, 2009–2010, 2011–2012, 2013–2014, and 2015–2016. Participants included were adults aged 20 years or older diagnosed with DM. Diagnosis was confirmed if participants met any of the following criteria: a physician's diagnosis of diabetes, current use of oral diabetic medication or insulin, a glycated hemoglobin (HbA1c) level exceeding 6.4%, a fasting glucose level over 125 mg/dL, or a 2-h glucose level of at least 200 mg/dL during an oral glucose tolerance test (OGTT).

Exclusion criteria included missing covariate data, absence of retinopathy questionnaire data, incomplete data necessary for calculating the CONUT index, or unavailability of sample weights.

### 2.4. Study Outcome

Retinopathy was determined through responses to the NHANES questionnaire: “Has a doctor ever told you that diabetes has affected your eyes or that you had retinopathy?” Participants were categorized as having retinopathy if they answered “yes.”

### 2.5. Assessment of CONUT

The CONUT score was selected for this study due to its ability to objectively reflect multiple aspects of nutritional status—protein reserves, immune competence, and lipid metabolism—through serum albumin, lymphocyte count, and cholesterol levels, respectively [8, 9]. Compared to other indices such as the Prognostic Nutritional Index (PNI) or Subjective Global Assessment (SGA), the CONUT score relies solely on routine laboratory values—serum albumin, total cholesterol, and lymphocyte count—which makes it relatively easy to calculate and less dependent on clinician judgment [14, 15]. While PNI also uses lab data, CONUT incorporates lipid metabolism and immune status in addition to protein reserves. Unlike SGA, which requires subjective clinical evaluation, CONUT offers greater standardization and is better suited for large-scale database studies such as NHANES.

The CONUT score was calculated using three parameters: serum albumin concentration, total lymphocyte count, and total cholesterol levels [8, 9]. Serum albumin levels were scored as 0 points for ≥ 3.50 g/dL, 2 points for 3.00–3.49 g/dL, 4 points for 2.50–2.99 g/dL, and 6 points for levels below 2.50 g/dL. Total lymphocyte counts receive 0 points if ≥ 1.6 (per 1000/mm^3^), 1 point for 1.2–1.599, 2 points for 0.8–1.199, and 3 points for counts below 0.8. Total cholesterol levels were assigned 0 points for concentrations ≥ 180 mg/dL, 1 point for 140–179 mg/dL, 2 points for 100–139 mg/dL, and 3 points for levels below 100 mg/dL. The sum of these values constitutes the total CONUT score, which classifies nutritional status into normal (0–1 points), light malnutrition (2–4 points), moderate malnutrition (5–8 points), or severe malnutrition (9–12 points), with scores of 2 or above indicating high nutritional risk.

### 2.6. Covariates

Demographic data: Age, sex, race, and education level were collected through in-person interviews using the NHANES family and sample person demographics questionnaires, facilitated by trained interviewers via the computer-assisted personal interviewing (CAPI) system (Confirmit Corp., New York, United States). The data were appropriately weighted according to NHANES protocols.

Health status and lifestyle assessment: Duration of diabetes was derived by subtracting the age at diabetes diagnosis from the participant's current age, with categorization into three groups: < 3 years, 3–10 years, and > 10 years.

Smoking status was classified based on participant responses into never, former, or current smoker. Alcohol consumption was considered excessive for participants reporting consumption of four or more alcoholic drinks per week.

Dietary intake was evaluated to identify individuals with low energy intake (< 25 kcal/kg/day) and low protein intake (< 0.6 g/kg/day) according to the 24-h dietary recall data collected by the NHANES.

Major comorbidities: Hypertension was confirmed if participants had been informed of high blood pressure on two or more occasions, were taking medication to reduce blood pressure, or had a measured systolic blood pressure of ≥ 140 mmHg or diastolic blood pressure of ≥ 90 mmHg. Chronic respiratory diseases included diagnosed asthma, chronic bronchitis, or emphysema. Cardiovascular disease (CVD) history was noted for any occurrences of coronary heart disease, angina, heart attack, stroke, or congestive heart failure. Chronic kidney disease (CKD) was defined as having a glomerular filtration rate (GFR) less than 60 mL/min/1.73 m^2^, calculated with the modified equation: 175 × (serum creatinine)^−1.154^ × (age)^−0.203^ × (0.742 if female) × (1.212 if African American).

Laboratory measurements: Key laboratory parameters included albumin, lymphocyte count, total cholesterol, triglycerides, HbA1c, blood urea nitrogen (BUN), and uric acid. These measurements were crucial for calculating the CONUT score and assessing the overall health status of the study participants.

### 2.7. Statistical Analysis

NHANES uses a complex, multistage, probability cluster sampling design to assure national representation, wherein sampling weights (WTSAF2YR), pseudostratum (SDMVSTRA), and pseudocluster (SDMVPSU) provided by NHANES were applied in all analyses as guided by the NCHS. Continuous variables are presented in weighted mean and standard error; categorical variables are presented in unweighted number and weighted proportion. To account for the complex design, as recommended by the NHANES guidelines, we used SAS SURVEY procedures, which allow for correct estimation from complex samples. Specifically, *p* values for group comparisons were calculated using PROC SURVEYFREQ for categorical data and PROC SURVEYREG for continuous data. While not direct equivalents to the chi-square test and independent *t*-test, these procedures serve the same purpose and are well suited for analyzing complex survey data. Under the same context, logistic regression models were performed with PROC SURVEYLOGISTIC to determine the association between the study variables and retinopathy. These are standard methodologies in various published studies using the NHANES data, ensuring that our statistical analysis is valid and robust. Multivariable regression analysis was adjusted for hypertension, CVD, CKD, and HbA1c. These covariates were selected based on clinical relevance and prior knowledge to achieve an appropriate balance between confounding control and model parsimony while avoiding potential overadjustment. Stratified analysis was performed to determine the moderators of the association between CONUT score and retinopathy. A two-sided *p* value of < 0.05 was regarded as statistically significant. All statistical analyses were performed using SAS statistical software (Version 9.4, SAS Inc., Cary, North Carolina, United States). All data were analyzed in 2024.

## 3. Results

### 3.1. Study Population

**Figure 1:**
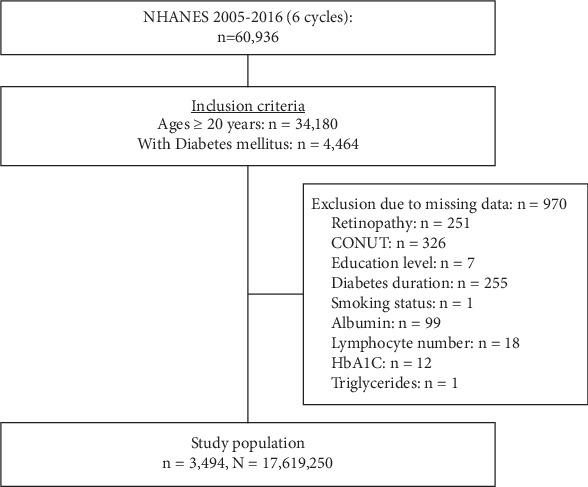
Flow diagram of study population selection. A total of 60,936 participants were identified in the NHANES during 2005–2016. Of these, 4464 adults aged 20 years and older with DM were initially considered. Exclusions were made for 970 patients lacking complete retinopathy questionnaire data or missing crucial components for the CONUT score calculation (i.e., serum albumin, total lymphocyte count, and total cholesterol), as well as information on education level, duration of diabetes, smoking status, HbA1c, and triglyceride levels. Consequently, 3494 participants were eligible for further analysis.

A total of 60,936 participants were identified in the NHANES during 2005 to 2016. Of these, 4464 adults aged 20 years and older with DM were initially considered. Exclusions were made for 970 patients lacking complete retinopathy questionnaire data or missing crucial components for the CONUT score calculation (i.e., serum albumin, total lymphocyte count, and total cholesterol), as well as information on education level, duration of diabetes, smoking status, HbA1c, and triglyceride levels. Consequently, 3494 participants were eligible for further analysis. This study sample was representative of an estimated 17,619,250 community-dwelling individuals in the entire United States using sample weights provided by the NHANES (Figure 1).

### 3.2. Study Population Characteristics

The mean age of the study sample was 59.7 years. 50.2% of the participants had attained an educational level beyond high school. Additionally, 15.0% of the participants had been living with DM for more than 10 years, and the most prevalent comorbidity was hypertension, affecting 72.3% of the study population. Notably, participants diagnosed with retinopathy exhibited higher CONUT scores compared to those without retinopathy (1.3 vs. 1.1, *p* value = 0.005), as shown in Table 1.

### 3.3. Associations Between CONUT Score and Retinopathy

The relationship between the CONUT score and the presence of retinopathy is summarized in Table 2. In the multivariable analysis, which adjusted for relevant confounding factors, a higher CONUT score (increase of 1 point) was significantly associated with increased odds of having retinopathy (adjusted odds ratio [aOR] = 1.11, 95% confidence interval [16]: 1.02–1.22, *p* value = 0.018) (Table 2).

### 3.4. Association Between CONUT and Retinopathy, Stratified by Diabetes Duration, Hypertension, and Age Group

Stratified analyses were conducted based on the duration of DM, presence of comorbid hypertension, and age groups, with the results presented in Table 3. When the CONUT score was evaluated as a continuous variable, it was significantly associated with increased odds of DR among patients with a diabetes duration ≥ 10 years (aOR = 1.14, 95% CI: 1.03–1.26, *p* value = 0.014), but this association was not observed in patients with DM duration < 10 years.

Among patients younger than 60 years, a higher CONUT score was significantly associated with greater odds of DR (aOR = 1.19, 95% CI: 1.03–1.37, *p* value = 0.016). Furthermore, a CONUT score ≥ 2 was significantly linked with increased odds of DR compared to scores less than 2 (aOR = 1.57, 95% CI: 1.05–2.35, *p* value = 0.028). However, the presence or absence of hypertension did not significantly influence the association between the CONUT score and retinopathy (Table 3).

## 4. Discussion

While DM affects 8.8% of adults worldwide and is expected to rise to 9.9% by 2040, actions such as medication adherence, dietary regulations, regular exercise, and consistent serum glucose monitoring can mitigate severe complications, including DR [2–5]. Meanwhile, the specific role of the CONUT score in patients with DR has not been clearly established, despite a strong rationale for its potential applications in various medical conditions [16–18]. Consequently, this study was designed to explore the relationship between the CONUT score and DR, based on a hypothesis that a higher CONUT score, corresponding to an inferior nutritional status, would be associated with an elevated likelihood of retinopathy in patients with DM. Our analysis reveals that higher CONUT scores, indicating worse nutritional status, are associated with a greater likelihood of DR after adjusting for relevant confounders in the multivariable analysis. Interestingly, the stratified analysis by age and diabetic duration revealed that this link is only significant in the subgroups of patients who have had diabetes for 10 years or more, as well as in younger patients under the age of 60.

Being the most prevalent microvascular complication of diabetes, DR is a leading cause of preventable blindness among adults [19]. It typically progresses without symptoms in its early stages, only becoming apparent when vision loss occurs in the advanced stages of the disease [20]. The progression of DR varies greatly among individuals, influenced by a mix of modifiable and nonmodifiable risk factors [21]. Due to the current inability to predict which diabetes patients will develop DR or the rate at which it will progress, clinical guidelines universally recommend annual DR screenings for all diabetic individuals without DR or with only mild DR [22, 23]. This provides the ground for identifying effective biomarkers for the early diagnosis of DR.

Specific to Type 2 DM, its comorbidities, complications, and mortality, a few studies have assessed the clinical relevance of the CONUT score over the past several years. For example, utilizing NHANES data accumulated over the years 1999–2018 from 3763 adult patients with T2D, one research group found recently that higher CONUT scores correlated with elevated mortality overall in individuals with T2DM [24]. Another recent analysis elucidated a similar result, with moderate and severe malnutrition based on CONUT scores being associated with elevated risks of all-cause death, and also revealed correlations between high CONUT scores and diabetic nephropathy-related death [25]. Additional studies have documented that nutritional status, as indicated by the CONUT score, is associated with worsened diabetic comorbidities and complications, including carotid atherosclerosis [26] and diabetic foot in those 75 years of age or older [27]. Also based on NHANES data obtained from 2011 to 2020, another analysis established a negative connection between serum albumin (a component of the CONUT score) and DR in older age groups, which is partly compatible with our findings [28]. While these prior studies support the prognostic value of the CONUT score or its components in diabetic populations, few have focused specifically on DR. Our analysis adds new insight by linking the CONUT score to retinopathy risk, particularly in younger patients and those with longer diabetes duration.

Intriguingly, our stratified analysis revealed a pronounced relationship between high CONUT score and the presence of retinopathy in patients under 60 years old, but not in older patients. A possible explanation is that the aging process introduces multiple comorbidities and metabolic changes, which the NHANES dataset did not fully account for, potentially masking the association between CONUT and retinopathy. Additionally, a significant association between the CONUT score and DR was observed only in patients with a diabetes duration of 10 years or more, but not in those with a shorter disease course. This pattern likely reflects the cumulative nature of microvascular damage resulting from prolonged hyperglycemia and systemic inflammation. Over time, nutritional and metabolic imbalances—captured by the CONUT score through markers such as hypoalbuminemia, immune dysfunction, and dyslipidemia—may further compromise vascular integrity and impair repair mechanisms, thereby contributing to the development and progression of retinopathy [6, 29]. It is known that within the first 10 years of diabetes, the prevalence of retinopathy is low and its progression is infrequent [30]. This highlights the importance of future prospective studies, which are strongly recommended to capture such nuanced changes over time more effectively.

In summary, although causal inference is limited by the cross-sectional design of this study, the findings highlight the potential importance of regular nutritional assessment in the comprehensive care of patients with diabetes. Given its simplicity and use of routinely available laboratory parameters, the CONUT score may serve as a practical tool for risk stratification to help identify individuals at increased risk of complications such as DR.

### 4.1. Strength and Limitation

This present study's strengths include the use of the comprehensive and nationally representative NHANES data, which enhance the generalizability of results across the US adult population. The rigor of the NHANES data collection methods and the application of the CONUT score, a standardized tool for assessing nutritional status, strengthen the study's analytical foundation. Nevertheless, limitations, such as the cross-sectional design, prevent causal inferences between nutritional status and DR. Furthermore, reliance on self-reported physician diagnoses for DR may introduce recall bias and potential misclassification, as the NHANES cycles analyzed did not include objective ophthalmologic assessments such as retinal imaging or fundus examinations. Additionally, potential biases resulting from reliance on self-reported diagnoses for several health conditions and residual confounding from unmeasured variables could affect the findings. Future studies, if feasible, should consider utilizing datasets that incorporate objective ophthalmologic assessments and adopt prospective longitudinal designs to better establish temporal relationships and causal pathways. These limitations emphasize the need for cautious interpretation of the observed associations and highlight the importance of future longitudinal studies to clarify causal relationships and assess the potential benefits of nutritional interventions. Although evaluating whether CONUT offers advantages over other nutritional indices was beyond the scope of this study, we agree that this remains an important area for future research.

## 5. Conclusion

This study found a significant association between higher CONUT scores and the presence of retinopathy among US adults with diabetes. The research emphasizes the importance of nutritional status in diabetes care. Future research should focus on longitudinal studies to explore causal relationships and the effectiveness of nutritional interventions in reducing complications of diabetes.

## Figures and Tables

**Table 1:** Characteristics of the of study population, categorized by retinopathy status.

**Characteristics**	**Total** **n** = 3494	**Retinopathy**		**p** ** value**
		**Yes**	**No**	
		**n** = 778	**n** = 2716	
CONUT	1.1 ± 0.03	1.3 ± 0.06	1.1 ± 0.03	**0.005**
High (≥ 2)	1029 (28.8)	266 (34.2)	763 (27.4)	**0.017**
Low (< 2)	2465 (71.2)	512 (65.8)	1953 (72.6)	
Demography				
Age (years)	59.7 ± 0.3	59.7 ± 0.6	59.7 ± 0.3	0.964
20–39	219 (7.8)	43 (6.8)	176 (8.1)	0.149
40–59	1097 (38.3)	255 (42.6)	842 (37.2)	
60–79	1874 (46.6)	415 (42.8)	1459 (47.6)	
80+	304 (7.2)	65 (7.8)	239 (7.1)	
Sex				0.638
Male	1800 (51.1)	418 (52.2)	1382 (50.9)	
Female	1694 (48.9)	360 (47.8)	1334 (49.1)	
Race				0.070
Non-Hispanic White	1217 (61.2)	250 (56.9)	967 (62.3)	
Non-Hispanic Black	934 (15.4)	210 (17.4)	724 (14.9)	
Hispanic	365 (5.7)	97 (6.1)	268 (5.6)	
Other	978 (17.7)	221 (19.6)	757 (17.3)	
Education level				**< 0.001**
< High school	1284 (25.6)	305 (30.4)	979 (24.4)	
High school	790 (24.2)	183 (27.4)	607 (23.3)	
> High school	1420 (50.2)	290 (42.1)	1130 (52.3)	
DM duration				**< 0.001**
< 3 years	490 (15.0)	59 (7.2)	431 (17.0)	
3–10 years	1442 (42.3)	247 (34.3)	1195 (44.3)	
> 10 years	1562 (42.7)	472 (58.4)	1090 (38.7)	
Smoking status				0.826
Never	1737 (49.4)	375 (49.9)	1362 (49.3)	
Former	1207 (34.7)	288 (35.1)	919 (34.6)	
Current	550 (15.8)	115 (15.0)	435 (16.1)	
Excessive alcohol consumption	149 (4.6)	27 (4.0)	122 (4.8)	0.570
Low energy intake (< 25 kcal/kg/day)	2272 (64.8)	503 (64.9)	1769 (64.7)	0.968
Low protein intake (< 0.6 g/kg/day)	986 (27.3)	238 (28.4)	748 (27.0)	0.559
Major comorbidities				
Hypertension	2595 (72.3)	611 (76.1)	1984 (71.3)	**0.020**
Chronic respiratory disease	673 (19.7)	163 (20.2)	510 (19.6)	0.720
CVD history	979 (26.5)	279 (35.2)	700 (24.4)	**< 0.001**
CKD	863 (23.3)	273 (34.1)	590 (20.6)	**< 0.001**
Laboratory measures				
Albumin (g/dL)	4.2 ± 0.01	4.1 ± 0.02	4.2 ± 0.01	**< 0.001**
Lymphocyte number (1000 cells/μL)	2.2 ± 0.03	2.1 ± 0.04	2.2 ± 0.04	**0.018**
Total cholesterol (mg/dL)	180.6 ± 1.2	181.2 ± 2.4	180.5 ± 1.4	0.810
Triglycerides (mmol/L)	2.2 ± 0.05	2.2 ± 0.09	2.2 ± 0.05	0.909
HbA1c (%)	7.4 ± 0.04	7.8 ± 0.08	7.3 ± 0.04	**< 0.001**
Blood urea nitrogen (mmol/L)	5.9 ± 0.07	7.0 ± 0.19	5.6 ± 0.06	**< 0.001**
Uric acid (μmol/L)	341.1 ± 2.5	349.8 ± 5.6	338.9 ± 2.62	0.070

*Note: p* values < 0.05 are shown in bold. Continuous variables are presented as mean ± SE. Categorical variables are presented as unweighted counts (weighted percentage).

**Table 2:** Associations between CONUT and the presence of retinopathy in patients with DM.

	**Retinopathy**			
	**OR (95% CI)**	**p** ** value**	**aOR ** ^**a**^ ** (95% CI)**	**p** ** value**
CONUT (continuous)	**1.15 (1.05–1.25)**	**0.004**	**1.11 (1.02–1.22)**	**0.018**
CONUT (high vs. low)	**1.38 (1.05–1.80)**	**0.020**	1.27 (0.98–1.64)	0.071

*Note: p* values < 0.05 are shown in bold.

Abbreviations: aOR, adjusted odds ratio; CI, confidence interval; CKD, chronic kidney disease; CONUT, Controlling Nutritional Status; CVD, cardiovascular disease; DM, diabetes mellitus; HbA1c, glycated hemoglobin; OR, odds ratio.

^a^Adjusted for hypertension, CVD, CKD, and HbA1c.

**Table 3:** Associations between CONUT and the presence of retinopathy, stratified by DM duration, hypertension, and age.

**Subgroup**	**Retinopathy**	
	**aOR ** ^**a**^ ** (95% CI)**	**p** ** value**
DM duration < 10 years		
CONUT (continuous)	0.97 (0.81–1.15)	0.685
CONUT (high vs. low)	0.97 (0.62–1.49)	0.869
DM duration ≥ 10 years		
CONUT (continuous)	**1.14 (1.03–1.26)**	**0.014**
CONUT (high vs. low)	1.37 (0.98–1.91)	0.064
No hypertension		
CONUT (continuous)	1.13 (0.93–1.36)	0.215
CONUT (high vs. low)	1.23 (0.69–2.20)	0.479
With hypertension		
CONUT (continuous)	1.10 (0.995–1.23)	0.063
CONUT (high vs. low)	1.28 (0.97–1.70)	0.083
Age < 60 years		
CONUT (continuous)	**1.19 (1.03–1.37)**	**0.016**
CONUT (high vs. low)	**1.57 (1.05–2.35)**	**0.028**
Age ≥ 60 years		
CONUT (continuous)	1.09 (0.98–1.22)	0.128
CONUT (high vs. low)	1.18 (0.86–1.62)	0.289

*Note: p* value < 0.05 were shown in bold.

Abbreviations: aOR, adjusted odds ratio; CI, confidence interval.

^a^Adjusted for hypertension, CVD, CKD, and HbA1c (except for the stratified variables).

## Data Availability

The datasets used and/or analyzed during the current study are available from the corresponding author upon reasonable request.
